# A Rare Diagnosis of Dubin‐Johnson Syndrome During Pregnancy: A Case Report

**DOI:** 10.1002/ccr3.73267

**Published:** 2026-08-06

**Authors:** Said Reza Modares Mousavi, Farab Pourhasan, Pouria Ahmadi Simab, Sepideh Beikmohammadi‐Gharehsaghghal, Daryoush Kaini‐Shamsabadi, Elaheh Karimzadeh‐Soureshjani

**Affiliations:** ^1^ Internal Medicine Department Abadan University of Medical Sciences Abadan Iran; ^2^ Department of Clinical Sciences Abadan University of Medical Sciences Abadan Iran; ^3^ Student of Research Committee, Abadan University of Medical Sciences Abadan Iran; ^4^ Universal Scientific Education and Research Network (USERN), Tehran Iran

**Keywords:** *ABCC2* gene, Dubin‐Johnson syndrome, genetic testing, hyperbilirubinemia, icterus, pregnancy

## Abstract

Dubin‐Johnson Syndrome (DJS) is a rare inherited disorder characterized by isolated conjugated hyperbilirubinemia without significant liver damage. This syndrome is often diagnosed incidentally during routine blood tests, as it typically presents with minimal or no symptoms. The disease results from a genetic defect in the *ABCC2* gene, which encodes the MRP2 protein responsible for transporting conjugated bilirubin out of hepatocytes. In individuals with DJS, the impaired function of MRP2 leads to the accumulation of conjugated bilirubin in the liver and bloodstream. This case report presents a 31‐year‐old female who, during pregnancy, developed icterus, which raised concerns about possible liver disease or viral hepatitis. However, further investigation revealed no significant liver dysfunction or risk factors for viral hepatitis. Laboratory tests showed elevated total and direct bilirubin levels, with normal liver enzymes, thus prompting further evaluation. Ultrasound imaging of the liver and biliary system revealed no signs of obstruction, and tests for viral hepatitis were negative. The patient's clinical history of icterus since childhood, combined with the laboratory findings, led to the suspicion of DJS. Notably, urine coproporphyrin analysis demonstrated a characteristic increase in coproporphyrin I, further supporting the diagnosis. Although DJS does not typically require treatment, the patient was provided with genetic counseling to inform her and her family about the condition and potential inheritance patterns. DJS presents a diagnostic challenge due to its subtle clinical features and the need to differentiate it from other causes of icterus, especially in pregnancy. This case highlights the importance of considering rare genetic disorders in the differential diagnosis of icterus and emphasizes the role of genetic testing in confirming the diagnosis. Early recognition of DJS allows for appropriate management and genetic counseling, ensuring that affected individuals can lead a normal life without major complications.

## Key Clinical Message

This case highlights Dubin‐Johnson syndrome (DJS) in a pregnant woman with lifelong jaundice, direct hyperbilirubinemia, and normal liver enzymes. DJS, a benign inherited disorder caused by ABCC2/MRP2 mutations, must be differentiated from obstetric liver diseases. No treatment is needed, but genetic counseling is advised. Accurate diagnosis prevents unnecessary interventions.

## 1 Introduction

In 1954, Dubin and Johnson, as well as independently, Sprinz and Nelson, described patients with chronic conjugated hyperbilirubinemia that was not associated with hemolysis. This disorder occurs in both men and women of all races and nationalities [1]. The condition became known as Dubin‐Johnson syndrome (DJS), a relatively rare hereditary form of hyperbilirubinemia characterized by a mild increase in conjugated bilirubin without other signs of liver damage. The disease results from a mutation leading to improper bilirubin excretion from hepatocytes. In most cases, DJS manifests during the second decade of life, though it may rarely appear in neonates [2].

Clinically, Dubin‐Johnson syndrome is characterized by mild icterus. Otherwise, patients remain asymptomatic, although mild complaints such as vague abdominal pain and fatigue may be present. Icterus can be so subtle that it is only noticed during concurrent illnesses, pregnancy, or the use of oral contraceptives. Patients do not experience pruritus. Physical examination is generally unremarkable except for icterus, although hepatosplenomegaly may occasionally be observed [1]. These patients often undergo extensive diagnostic evaluations for unexplained icterus and experience significant anxiety.

One of the hallmark features of DJS is the accumulation of coarse, dark, granular pigment within the lysosomes of centrilobular hepatocytes. As a result, the liver may take on a strikingly black appearance. This pigment is believed to be derived from epinephrine metabolites that are not properly excreted. In comparison with findings from several mutant mouse strains, the selective defect in biliary excretion of conjugated bilirubin and specific classes of organic compounds (but not bile acids) characteristic of DJS in humans suggests defective expression of MRP2 (*ABCC2*), an ATP‐dependent canalicular membrane transporter. Various mutations in the *ABCC2* gene give rise to the Dubin‐Johnson phenotype, which follows an autosomal recessive inheritance pattern. Although MRP2 plays a crucial role in the biliary excretion of conjugated bilirubin, the fact that bilirubin excretion still occurs in the absence of MRP2 suggests that other, yet unidentified, transport proteins may have a secondary role in this process [3].

Routine clinical laboratory tests, including complete blood count, serum albumin, cholesterol, alanine and aspartate aminotransferases, alkaline phosphatase, and prothrombin time, are typically normal. Both fasting and postprandial bile acid levels, which serve as sensitive markers of liver disease, remain within normal ranges, indicating that the genetic defect does not affect bile acid transport [1].

Despite its benign nature, DJS is often misdiagnosed as other hepatobiliary disorders, leading to unnecessary investigations and patient anxiety. The black discoloration of the liver, although a distinguishing pathological feature, is not evident in routine imaging studies, making diagnosis challenging. Given the rarity of this condition and its potential diagnostic confusion with more serious hepatic pathologies, reporting rare cases of DJS remains clinically valuable. In this study, we present a rare case of Dubin‐Johnson syndrome, highlighting its clinical presentation, diagnostic approach, and potential challenges in differentiation from other causes of hyperbilirubinemia. This case adds to the existing literature by providing further insight into the clinical spectrum of DJS and its diagnostic considerations.

## 2 Case History/Examination

A 31‐year‐old woman (G4P2A1) was admitted for preterm labor and delivered vaginally. She presented with longstanding jaundice since childhood, with icteric sclera and skin, and had known thalassemia minor. There was no history of fever, biliary pain, hepatitis symptoms, or risk factors for infectious hepatitis. Physical exam revealed no signs of chronic liver disease, with normal abdominal findings except for mild splenomegaly (168 mm) on ultrasound. Initial labs showed marked direct hyperbilirubinemia (total bilirubin 9.2 mg/dL, direct 6.4 mg/dL) with normal AST/ALT, elevated ALP (498 IU/L), normal PT/INR, and 3+ bilirubin in urinalysis (Tables 1, 2). Imaging showed normal liver architecture without biliary obstruction.

**TABLE 1:** Initial laboratory findings.

Test	Result	Unit	Normal range
Total bilirubin	9.2	mg/dL	0.1–1.1
Direct bilirubin	6.4	mg/dL	0–0.2
AST	35	IU/L	0–31
ALT	23	IU/L	0–32
ALP	498	IU/L	80–290
PTT	32	sec	24–36
PT	11	sec	13–15
INR	1	—	1–1.16

*Note:* Urinalysis showed bilirubin 3 +.

Abbreviations: ALP, alkaline phosphatase; ALT, alanine aminotransferase; AST, aspartate aminotransferase; INR, international normalized ratio; PT, prothrombin time; PTT, partial thromboplastin time.

**TABLE 2:** Follow‐up laboratory findings.

Test	Result	Unit	Normal range
Total bilirubin	9.9	mg/dL	0.1–1.1
Direct bilirubin	9.4	mg/dL	0–0.2
AST	26	IU/L	0–31
ALT	23	IU/L	0–32
ALP	394	IU/L	80–290
Serum LDH	402	IU/L	235–470
Total Urine coproporphyrin	15	μg/24 h	< 60
Urine coproporphyrin I	12.8	μg/24 h	—

Abbreviations: ALP, alkaline phosphatase; ALT, alanine aminotransferase; AST, aspartate aminotransferase; Serum LDH, serum lactate dehydrogenase.

## 3 Differential Diagnosis, Investigations and Treatment

The differential diagnosis centered on inherited hyperbilirubinemia syndromes, particularly Dubin‐Johnson Syndrome (DJS) given the isolated direct hyperbilirubinemia, normal liver enzymes, and chronic history. Rotor syndrome, hemolysis, biliary obstruction, and viral hepatitis were considered but excluded based on clinical and laboratory findings. Diagnostic evaluation included urinary coproporphyrin analysis which showed elevated coproporphyrin I (12.8 μg/24 h, > 80% of total), confirming DJS. No specific treatment was required for this benign condition, with management focused on patient reassurance and genetic counseling.

## 4 Outcome and Follow‐Up

The patient was definitively diagnosed with Dubin–Johnson syndrome, an autosomal recessive benign disorder of bilirubin metabolism. At two‐month follow‐up, total bilirubin remained stable (7.25 mg/dL) without evidence of hepatic dysfunction (Table 3). No long‐term monitoring was necessary.

**TABLE 3:** Follow‐up laboratory findings (2 months later).

Test	Result	Unit	Normal range
Total bilirubin	7.25	mg/dL	0.1–1.1
Direct bilirubin	5.97	mg/dL	Up to 0.7
AST	29	IU/L	5–40
ALT	39	IU/L	5–40
ALP	248	IU/L	Up to 258
Albumin	4.38	g/dL	3.8–5.1
PT	12.5	sec	10–13
INR	1	—	1
Gamma GT	35	U/L	Up to 39

Abbreviations: Albumin, serum albumin; ALP, alkaline phosphatase; ALT, alanine aminotransferase; AST, aspartate aminotransferase; Gamma‐GT, gamma‐glutamyl transferase; INR, international normalized ratio; PT, prothrombin time.

This case demonstrates that DJS can be reliably diagnosed even in the complex physiological context of pregnancy and the postpartum period, where biochemical alterations may complicate interpretation. It underscores the diagnostic value of urinary coproporphyrin analysis in distinguishing benign hereditary hyperbilirubinemia from serious pregnancy‐related hepatobiliary disorders, thereby preventing unnecessary investigations and interventions while enabling appropriate counseling. The patient's prognosis remains excellent, with no anticipated long‐term complications.

## 5 Discussion

The prevalence of Dubin‐Johnson Syndrome (DJS) is estimated to be less than one case per 100,000 people worldwide, but it is more common among Iranian Jews. Although this disease is considered rare worldwide, reports show that its incidence among Sephardic Jews is approximately 1 in 3000. DJS is typically seen in young adults without symptoms, although hyperbilirubinemia is often diagnosed incidentally during routine tests. In rare cases, mild symptoms such as jaundice, fatigue, and/or abdominal pain may occur. Unlike other conditions that cause hyperbilirubinemia (such as liver diseases), DJS usually does not cause itching because serum bile acids are normal. In some cases, women with this disease may have no symptoms, but hyperbilirubinemia or jaundice may become apparent with the use of oral contraceptives or pregnancy. The differential diagnosis of jaundice during pregnancy includes viral hepatitis, intrahepatic cholestasis of pregnancy, acute fatty liver of pregnancy, HELLP syndrome (Hemolysis, Elevated Liver Enzymes, and Low Platelets), and rarely hereditary disorders like Dubin‐Johnson syndrome and Rotor syndrome. Viral hepatitis is the most common cause of jaundice in pregnancy. A history of arthralgia and myalgia before jaundice suggests hepatitis [4, 5]. Patients with acute viral hepatitis typically have aminotransferase levels above 500 IU/L, with alanine aminotransferase (ALT) equal to or greater than aspartate aminotransferase (AST) [6].

Intrahepatic cholestasis of pregnancy (ICP), also known as obstetric cholestasis, is a liver disease during pregnancy associated with elevated serum bile acids and increased adverse fetal outcomes. The most common symptom of ICP is itching, especially in the third trimester. This itching progressively worsens during pregnancy and usually improves 48 h after delivery. In ICP, ALT and AST may increase before or after bile acids. Among these enzymes, ALT is considered a more sensitive marker for ICP. Typically, there is a 2‐ to 10‐fold increase in serum ALT, which is generally greater than the increase in AST. Bilirubin is usually normal in most cases of ICP and has limited diagnostic or monitoring value. If bilirubin increases, it is often conjugated hyperbilirubinemia. Alkaline phosphatase (ALP) may be elevated in ICP, but the production of large amounts of isoforms from the placenta limits the diagnostic value of this marker. Measurement of serum bile acids is now considered the most appropriate biochemical marker for diagnosing and monitoring ICP. Reports of serum bile acid levels increasing up to 100 times the upper limit of normal have been found [7].

Acute fatty liver of pregnancy (AFLP) is an obstetric emergency characterized by liver dysfunction and/or failure, which can lead to maternal and fetal complications, including death. Rapid delivery and supportive care for the mother are critical to achieving full recovery for the mother. Initial symptoms of AFLP are often nonspecific (e.g., nausea, vomiting, abdominal pain, weakness, headache, and/or anorexia). Many patients also have hypertension, with or without proteinuria. Concurrent hemolysis, elevated liver enzymes, and low platelets occur in 20% of cases, and 20% to 40% of patients are also diagnosed with preeclampsia. All patients with AFLP have elevated aminotransferases (aspartate aminotransferase or alanine aminotransferase), typically between 5 and 10 times the normal level [8].

HELLP syndrome is a pregnancy‐related liver disorder that was first described by Weinstein in 1982 as a set of clinical and laboratory abnormalities in pregnant women in the third trimester. This disorder is called HELLP syndrome because (H) stands for hemolysis, (EL) stands for elevated liver tests, and (LP) stands for low platelets. Affected pregnant women usually present with nonspecific symptoms such as weight gain, right upper quadrant pain (90%), nausea or vomiting (50%), or nonspecific viral‐like illness. Jaundice occurs in 40% of patients. Signs and symptoms of concurrent preeclampsia may or may not be present. Pregnant women with preeclampsia/HELLP may present with involvement of other organs, including neurological or visual symptoms, pulmonary edema, and renal failure. Liver enzyme abnormalities occur in 10% of pregnant women with severe preeclampsia. In these cases, there is typically a 2‐ to 3‐fold increase in alanine and aspartate aminotransferases. The frequency and severity of elevated liver enzymes are much higher in HELLP syndrome than in severe preeclampsia. Hemolysis is one of the hallmark features of HELLP syndrome. The presence of an abnormal peripheral smear (microangiopathic hemolytic anemia with schistocytosis), thrombocytopenia, and elevated aspartate aminotransferase, alanine aminotransferase, bilirubin, and LDH levels are diagnostic for these diseases [9].

The diagnosis of Dubin‐Johnson syndrome is confirmed with conjugated hyperbilirubinemia (in which at least 50% of the total bilirubin is direct bilirubin) while the rest of the liver function profile (alanine aminotransferase, aspartate aminotransferase, alkaline phosphatase, albumin, and prothrombin time) is normal. Characteristic urinary coproporphyrin excretion also supports the diagnosis (total urinary coproporphyrin in Dubin‐Johnson syndrome is normal, but over 80% of it is coproporphyrin I, in contrast to normal individuals, in whom 75% of the urinary coproporphyrin is coproporphyrin III) [1].

In patients with mild hyperbilirubinemia (with approximately 50% direct fraction) in the absence of other abnormalities in standard liver function tests, Dubin‐Johnson syndrome and Rotor syndrome should be suspected. Normal serum alkaline phosphatase and gamma‐glutamyl transpeptidase levels help differentiate these conditions from obstructive biliary disorders. The pattern of urinary coproporphyrin excretion helps distinguish the two disorders. In Dubin‐Johnson syndrome, total urinary coproporphyrin is normal, but 80% of it is coproporphyrin I (in normal individuals, 75% of urinary porphyrins are coproporphyrin III). In contrast, in Rotor syndrome, total urinary coproporphyrins increase to 250 to 500% of normal, with approximately 65% being coproporphyrin I. Liver biopsy is not necessary to diagnose either disorder. However, if a biopsy is performed for another reason, the dense pigment seen in the liver in Dubin‐Johnson syndrome helps distinguish it from Rotor syndrome. Dubin‐Johnson syndrome is a benign condition and does not require treatment [1].

In DJS, the excretion of several anionic compounds is impaired. These compounds include various cholescintigraphic agents as well as bromsulphalein (BSP), a synthetic dye previously used in liver function tests. In this test, the rate of BSP disappearance from plasma after IV bolus injection was measured. BSP is conjugated with glutathione in hepatocytes. The conjugate is usually rapidly excreted in the bile ducts. Patients with DJS show a distinct increase in plasma concentration of this substance 90 min after injection due to the backflow of BSP conjugates from hepatic cells into the bloodstream [3]. Conjugated bilirubin cannot effectively pass through the placental and blood–brain barriers and is therefore unlikely to affect the fetus. Although unconjugated bilirubin can pass through the placenta and blood–brain barrier, its concentration in the fetus is likely too low to cause kernicterus in the absence of other adverse fetal events [10].

Mutations in the *ABCC2* gene, which helps produce the *MRP2* (*Multidrug Resistance Protein 2*), are the primary cause of Dubin‐Johnson Syndrome (DJS). *MRP2* is a membrane transporter in the liver responsible for exporting conjugated bilirubin from liver cells into the extracellular space and subsequently excreting it from the body [11]. When there is a mutation in the *ABCC2* gene, the function of *MRP2* is impaired, preventing conjugated bilirubin from leaving liver cells. This results in elevated levels of conjugated bilirubin in the blood, leading to hyperbilirubinemia, with jaundice being the most common clinical symptom. This disorder typically presents as mild and without severe clinical manifestations. Genetic and clinical studies have confirmed that mutations in *ABCC2* are the primary cause of DJS [12, 13, 14]. In this patient, laboratory tests showed increased conjugated bilirubin levels and defective *MRP2* function, which corroborates the diagnosis of DJS. It should be acknowledged that *ABCC2* gene analysis was not performed in this case. This was primarily due to limited laboratory resources and the high cost of genetic testing, which are common constraints in the present study. Although the condition is generally benign and the symptoms usually resolve on their own, careful monitoring and regular follow‐up are essential for assessing the patient's condition. DJS typically does not require specific treatment, as most patients manage the condition with periodic medical care without the need for complex interventions. However, if the disorder is found in other family members, genetic counseling is important to identify individuals at risk and assess the possibility of transmission to future generations. In cases where individuals are suspected of having DJS, genetic tests to identify mutations in the *ABCC2* gene and biochemical tests to measure bilirubin levels and other liver markers can assist in a more accurate diagnosis. Ultimately, while DJS is generally without serious complications, awareness of the disorder and monitoring the patient's status are crucial to prevent any future issues.

## Author Contributions

**Said Reza Modares Mousavi:** conceptualization, investigation, writing – original draft. **Farab Pourhasan:** data curation, formal analysis, writing – original draft. **Pouria Ahmadi Simab:** writing – original draft, writing – review and editing. **Sepideh Beikmohammadi‐Gharehsaghghal:** writing – original draft. **Daryoush Kaini‐Shamsabadi:** writing – original draft. **Elaheh Karimzadeh‐Soureshjani:** conceptualization, data curation.

## Funding

The authors have nothing to report.

## Ethics Statement

Ethical approval for this study was obtained from the Research Ethics Committee of Abadan University of Medical Sciences (IR.ABADANUMS.REC.1404.018). The study was conducted in accordance with the university's guidelines, ensuring the protection of patient rights and confidentiality. Informed consent was obtained from the patient's parent or their legal guardian, including consent for publication of the data in this article. The study was conducted in compliance with the ethical standards outlined in the 1964 Declaration of Helsinki and its subsequent amendments.

## Consent

Informed consent for publication of the data and the use of the patient's information in this article was obtained from the patient or their legal guardian. The consent form was signed by the patient's parent, who is the legal representative of the patient. The patient's identity has been kept confidential, and no personally identifiable information, including names, initials, addresses, or any other data that could identify the patient, is disclosed in the manuscript. All subjects gave their informed consent prior to their inclusion in the study, and any information that could reveal the identity of the patient was not published.

## Conflicts of Interest

The authors declare no conflicts of interest.

## Data Availability

The original data presented in the study are included in the article. For inquiries, please contact the corresponding author.
